# Cats are not small dogs: is there an immunological explanation for why cats are less affected by arthropod-borne disease than dogs?

**DOI:** 10.1186/s13071-016-1798-5

**Published:** 2016-09-20

**Authors:** Michael J. Day

**Affiliations:** School of Veterinary Sciences, University of Bristol, Langford, North Somerset, BS40 5DU UK

**Keywords:** Cat, Dog, Arthropod-borne infectious disease, Disease prevalence, Immune system, Immune function, Genetics, Microbiome

## Abstract

It is widely recognized that cats appear to be less frequently affected by arthropod-borne infectious diseases than dogs and share fewer zoonotic pathogens with man. This impression is supported by the relative lack of scientific publications related to feline vector-borne infections. This review explores the possible reasons for the difference between the two most common small companion animal species, including the hypothesis that cats might have a genetically-determined immunological resistance to arthropod vectors or the microparasites they transmit. A number of simple possibilities might account for the lower prevalence of these diseases in cats, including factors related to the lifestyle and behaviour of the cat, lesser spend on preventative healthcare for cats and reduced opportunities for research funding for these animals. The dog and cat have substantially similar immune system components, but differences in immune function might in part account for the markedly distinct prevalence and clinicopathological appearance of autoimmune, allergic, idiopathic inflammatory, immunodeficiency, neoplastic and infectious diseases in the two species. Cats have greater genetic diversity than dogs with much lower linkage disequilibrium in feline compared with canine breed groups. Immune function is intrinsically related to the nature of the intestinal microbiome and subtle differences between the canine and feline microbial populations might also impact on immune function and disease resistance. The reasons for the apparent lesser susceptibility of cats to arthropod-borne infectious diseases are likely to be complex, but warrant further investigation.

## Background

In recent years there has been renewed interest in investigating the epidemiology, clinicopathological mechanisms and phylogeny of the causative organisms of canine arthropod-borne infectious diseases. Molecular and immunological tools have allowed the discovery of novel pathogens, the reclassification of other microorganisms and provided the ability to undertake surveillance studies that track the geographical movement of these agents and their arthropod vectors. Some of these studies are performed from a ‘One Health’ perspective; with the recognition that many of the canine arthropod-borne infections are zoonotic or that the dog may act as a reservoir or sentinel for human infection [1]. As our companion dogs so closely share our lifestyle and indoor environment, there is recognition that control of these diseases must involve strategies to prevent infection in both people and dogs. One of the strongest cases for a One Health approach to these diseases is that of zoonotic visceral leishmaniosis, where prevention of human infection necessitates control of infection in the canine reservoir in addition to management of the sand fly vector [2].

But what of the other important small companion animal species - the domestic cat? The cat is ubiquitous in both developed and developing societies and equally shares the human environment with the dog [3]. Where numbers of small companion animals are estimated, it is clear that there are similar populations of pet dogs and cats living in human households [4–6] and in developing countries there are significant, but unquantified, populations of stray or community-owned dogs and cats. For example, in the USA in 2011 there were an estimated 69 million dogs living in 36.5 % of households and 74 million cats in 30.4 % of households [4]. In the UK in the same year, there were an estimated 11.5 million dogs in 30 % of households and 10 million cats in 23 % of households [6]. But despite the popularity of the cat as a companion animal, there is relatively little knowledge about the prevalence or nature of feline arthropod-borne infectious diseases. Recent reviews cover these infections [7, 8], but it is clear that we understand less about the same agents in cats compared with dogs. Anecdotally, it is often suggested that cats are less affected by arthropod-borne diseases than dogs and that this may be attributed to some form of natural resistance to these pathogens or their vectors. This would appear to be supported by a relatively low prevalence of most infections recorded in cats in areas in which the diseases are endemic [9–12]. The aim of this review is to explore this hypothesis and examine the evidence that underpins this proposal.

### Do cats get less arthropod-borne infectious disease and if so, why?

A recent study suggests that the number of zoonoses shared between man and different domestic animal species is determined by the time since that species was domesticated. The dog shares the most infectious diseases with people as dogs were first domesticated at least 15,000 years ago. In contrast, feline diseases shared with man are suggested to be only one third of the number of those of the dog because domestication of the cat occurred 10,000 years ago [13].

If one considers the prevalence of the major arthropod-borne infectious diseases of small companion animals, a ‘broad brush’ perspective would suggest that cats are less affected by these conditions than dogs (Table 1). To assess this in a somewhat more robust fashion, a search of the Thomson Reuters Web of Science database (performed in May 2016) was performed using the search terms ‘dog AND arthropod borne disease’, ‘cat AND arthropod borne disease’, ‘dog AND vector borne disease’ and ‘cat AND vector borne disease’. Between 1997 and May 2016, this search revealed 496 publications for the dog and 175 for the cat, with marked rises in the number of publications related to both species from 2008 onwards.

**Table 1:** Relative prevalence of canine and feline arthropod-borne infections

Infection	Dog	Cat	Recent reference for feline infection
Dirofilariosis	Common	Prevalence in cats ~10 % that in dogs	[104]
Babesiosis	Common	Uncommon (mostly in South Africa)	[105, 106]
Cytauxzoonosis	No	Yes	[107]
Haemotropic *Mycoplasma*	Problem only in splenectomized dogs	Common and clinically significant	[108]
Hepatozoonosis	Relatively common	Rare	[10]
Leishmaniosis	Common	Less common	[88, 89]
Borreliosis	Relatively common	Rare	[11, 109]
Bartonellosis	Less common?	Common	[110]
Ehrlichiosis	Relatively common	Rare	[111]
Anaplasmosis	Relatively common	Less common	[112]
Rickettsiosis	Relatively common	Less common	[113]

So what could be the reasons for the apparent difference in the prevalence of canine and feline arthropod-borne diseases? There are many possibilities and a number of these are far more pragmatic than the more interesting hypothesis of some form of natural resistance of the feline species to these diseases. The fewer publications may simply reflect the fact that less research is performed on the feline diseases, because there is less funding available for feline research and consequently there are fewer commercially available diagnostic tests or published research methodologies for the cat. The research community that focuses on feline arthropod-borne infectious diseases is much smaller than that which studies the equivalent disorders in the dog.

An alternative hypothesis might be that cats are simply taken for veterinary attention less often than dogs and the diseases are consequently less often diagnosed and recorded. Fewer available cases of a particular disease or infection makes it much more challenging to aquire a sufficient number of cases for a meaningful research investigation. Owner spend on preventative healthcare is thought to be less for cats than for dogs. For example, it has been suggested that cats are less frequently vaccinated than dogs. A UK survey of 3103 cat owners showed that 69 % of cats were currently vaccinated, but the survey likely selected for more dedicated cat owners [14]. In the USA, 81 % of dog-owning households made at least one veterinary visit in 2011 spending an average of $227 per dog, but only 55 % of cat owners sought veterinary attention, spending an average of $90 per cat [4]. Lesser preventative healthcare in turn may relate simply to the relatively independent nature and lifestyle of cats, the fact that cats are better able to ‘hide’ the signs of illness, the lesser value often placed on cats by society and the practical difficulties in transporting a cat for veterinary attention. Preventative healthcare veterinary visits also appear to decline with increasing age of the cat [15].

The feline lifestyle *per se* may also impact on the prevalence of arthropod-borne infections. In some countries, many more cats have an indoor only lifestyle that of course minimizes the risk of exposure to arthropods [14, 16]. But, even where cats have outdoor access, does their behaviour also limit arthropod exposure? Are cats better able to avoid questing ticks or sandfly bites or does their more fastidious grooming behaviour mean that they are likely to dislodge ticks before transmission of a microparasite? Or is it possible that cats have a natural chemical signal that provides resistance to arthropod bites as do individual humans [17]?

However, the most interesting hypothesis would be that cats have a natural, genetically controlled immunological resistance to arthropods and the microorganisms they transmit. Perhaps the feline immune system is less susceptible to the range of immunomodulatory salivary proteins contained within arthropod saliva [18–22] and the cat is more competent at generating protective or sterilizing immune responses to arthropod-borne pathogens. The remainder of this review will focus on the feline immune system and whether there are differences to that of the dog that might account for an apparent difference in susceptibilty to these pathogens.

### Are there differences between the canine and feline immune systems?

Only 30 years ago the study of canine and feline immunology was in its infancy, with few reagents and techniques limiting the ability to investigate humoral and cellular immune responses. The discovery of the feline immunodeficiency virus and the suggestion that the cat was an appropriate model for human immuodeficiency virus infection led to a period of research funding and development of immunological methods throughout the 1990s [23–25]. Shortly after there was similar development of reagents for canine immunology and interest in exploring canine immunogenetics and the association of canine diseases with genes of the major histocompatibility complex (MHC) [26–28]. The most significant breakthrough in canine immunology came with publication of the canine genome in 2005 [29], which enabled the rapid development of molecular means of detecting and characterizing a wide range of canine cytokines, chemokines, pattern recognition receptors and lymphocyte subsets. Similar methodology was developed for feline immunology, although the first complete feline genome was not published until 2014 [30].

Broadly assessing the published literature on canine and feline immunology, there are no simple significant differences between the two species [31]. Both species have the same range of lymphoid subsets, with T helper (Th) 1, Th2, Th17 and T regulatory (Treg) cell function indentified in each by expression of the same range of cytokines and key molecules such as forkhead box P3 (FoxP3; considered as a marker of Treg cells). Both species express the same range of pattern recognition receptors (Toll-like receptors, nucleotide-binding oligomerization domain containing [NOD]-like receptors and others) and have the same spectrum of antigen presenting cells. Less is known about phagocytic cell function and the complement pathways, although there is little reason to suspect any significant differences.

There may, however, be subtle differences in canine and feline immunoglobulins (Igs). The dog has four IgG subclasses which are functionally equivalent to those of man [32, 33]. In contrast, only three IgG subclasses are recognized in the cat [34]. Both species have IgM and IgE antibodies, although IgD has only been identified formally in the dog [35]. There may also be differences in IgA - both species have IgA, but in the dog four genetic variants of the molecule are reported [36], but there have been no equivalent studies of feline IgA.

### Do dogs and cats have different susceptibility to disease?

Although dogs and cats appear to have generally similar immune systems, there are distinct species differences in susceptibility to or clinical presentation of diseases that are caused by or that involve the immune system. This might suggest that although the components of the immune system are equivalent in both species, these components might interact differently, leading to distinct immunological outcomes.

Autoimmune diseases, in which the immune system reacts inappropriately against self tissue antigens, are multifactorial in pathogenesis, but involve immune imbalance - particularly with respect to impairment in the function of natural regulatory T cells [37]. A wide spectrum of autoimmune diseases is well documented and relatively common in the dog and these diseases often closely mimic the equivalent disorders in man [38, 39]. Canine autoimmune diseases are associated with autoantibodies and/or autoreactive cytotoxic T lymphocytes and reduced Treg function [39]. The diseases are breed-associated and often are familial and, like in people, there are clear links to the inheritance of particular susceptibility haplotypes of MHC genes [40]. In contrast, autoimmune diseases are relatively uncommon in the cat and there are no clear breed or familial associations, and no genetic basis is described.

Allergic diseases also present distinctly in dogs and cats. Cutaneous allergy is common in the dog (e.g. atopic dermatitis, flea allergy dermatitis) and food-associated allergy affecting the gastrointestinal tract is also increasingly recognized. However, allergic respiratory disease (i.e. eosinophilic bronchopneumopathy; EBP) is uncommon in dogs. Canine allergic diseases (particularly atopic dermatitis) are breed associated and familial [41–43] and are related to the function of induced regulatory T cells [44]. Again in contrast, feline allergy is relatively poorly defined and differs to the canine diseases in prevalence and presentation. Feline asthma is probably more common than canine EBP, yet atopic dermatitis may be more common in dogs compared with cats [45]. Feline cutaneous atopy has a spectrum of clinical presentation (the eosinophilic granuloma complex) that is distinctly different to the lesions of canine atopic dermatitis [46, 47].

Idiopathic inflammatory diseases affect both species, but again with some unique species differences. For example, both dogs and cats suffer from idiopathic inflammatory bowel disease (IBD); in particular, lymphoplasmacytic enteritis. In both species, the immunopathogenesis of IBD is proposed to reflect a combination of dysbiosis of the intestinal microbiome, intestinal barrier dysfunction and underlying immunological imbalance reflected in reduced activity of regulatory T cells permiting overactivity of Th1 and Th17 effector cells. However, there are differences in baseline intestinal immunity and in the immunopathology of IBD in dogs and cats. Cats have higher numbers of small intestinal intraepithelial lymphocytes than dogs [48, 49], but only canine enterocytes show consitutive expression of MHC class II molecules [49]. Dogs with IBD have a significant increase in the numbers of T cells and plasma cells infiltrating the intestinal lamina propria [50], which does not occur in cats [51]; however, cats with IBD have induced expression of MHC class II molecules on enterocytes. Cytokine gene expression studies within lesional tissue have consistently failed to demostrate differences between normal and inflamed canine intestine [52, 53], but in cats, increased expression of proinflammatory, Th1- and Treg-related cytokines has been shown [54]. Canine IBD more clearly has a genetic component with strong breed predispositions and genetic associations; for example the links between polymorphisms in Toll-like receptor genes and IBD in German shepherd dogs [55, 56]. No such associations are reported for feline IBD, but cats more frequently have concurrent hepatic and pancreatic inflammatory disease (‘triaditis’) than dogs [57]. Finally, although unproven, it has long been suggested that feline chronic intestinal inflammation may be a precursor to alimentary lymphoma [58], but this transition is less clearly recognized in the dog.

Primary inherited immunodeficiency diseases markedly differ between dogs and cats. In the dog, there is a spectrum of some 30 distinct breed-related putative immunodeficiency disorders, although only four of these have been characterized as to the genetic mutation responsible for the disease (i.e. the canine leucocyte adhesion deficiency, canine severe combined immunodeficiency [X-linked and not], the trapped neutrophil syndrome and the grey collie syndrome) [39, 59]. In distinct contrast, only three primary immunodeficiency diseases are reported in the cat: Pelger-Huet anomaly, Chediak-Higashi syndrome and a genetic mutation resulting in athymic and hairless Birman kittens [39, 60].

Dogs and cats also develop different spectra of neoplastic diseases and it is now clear that the immune system plays a crucial role in determining the biological behaviour of tumours - in particular, the effects of tumour-infiltrating Tregs and tumour-associated macrophages that impair anti-tumour immune responses and promote metastasis via tissue remodelling and neoangiogenesis [61–63]. With the recent availability of large cancer registries for both species [64, 65], we can now appreciate some of the species differences in the type, distribution and biological behaviour of canine and feline tumours. Although skin tumours are most commonly documented in both species, the relative occurrence of other neoplasms is not consistent between dogs and cats [64, 65]. Some examples to illustrate these differences would include: the feline injection site sarcoma [66] which is almost never reported in the dog, haemangiosarcoma which arises commonly in the spleen or heart of the dog [67] and only rarely in the skin of the cat, the spectum of histiocytic tumours of the dog [67, 68] which are almost unknown in the cat, the greater malignancy of feline compared with canine mammary tumours [69], but the relatively benign behaviour of feline versus canine cutaneous mast cell tumour [70]. There are again clear genetic associations for canine tumours (e.g. haemangiosarcomas in German shepherd dogs, histiocytic tumours in Bernese Mountain dogs and flat coated retrievers, mast cell tumours in Boxers and Labradors) [67] that are not recognized in the cat.

Finally, dogs and cats are susceptible to different spectra of infectious diseases other than the arthropod-borne infections. For example, dogs are more commonly affected by bacterial pyoderma [71], leptospirosis [72–74] and systemic or non-invasive upper respiratory fungal infections [75] than cats, but cats are increasingly reported with mycobacterial infections [76] or invasive upper respiratory tract fungal infections [77]. Cats are much more often affected by a range of viral infections than dogs (e.g. feline leukaemia virus, feline immunodeficiency virus, feline calicivirus, feline herpesvirus type 1 and feline infectious peritonitis virus). Despite the global occurrence of feline retrovirus infections there is no clear evidence for a canine retrovirus although endogenous canine retrovirus sequence within the canine genome shows that such viruses existed before the evolutionary divergence of the dog and the red fox [78, 79]. Cats appear susceptible to experimental infection with influenza viruses [80, 81] and the SARS coronavirus [82] and may sometimes develop clinical signs related to these infections, but this species is often considered relatively resistent to natural influenza virus infection. In contrast, dogs develop clinical disease when infected with influenza A viruses that originated in horses (H3N8 virus) or birds (H3N2 virus) [83]. Canine distemper virus and canine adenovirus infections involve other species, but not domestic cats [84, 85], while canine parvovirus type 2 appears to move back and forth between cats and dogs [86].

**Fig. 1:**
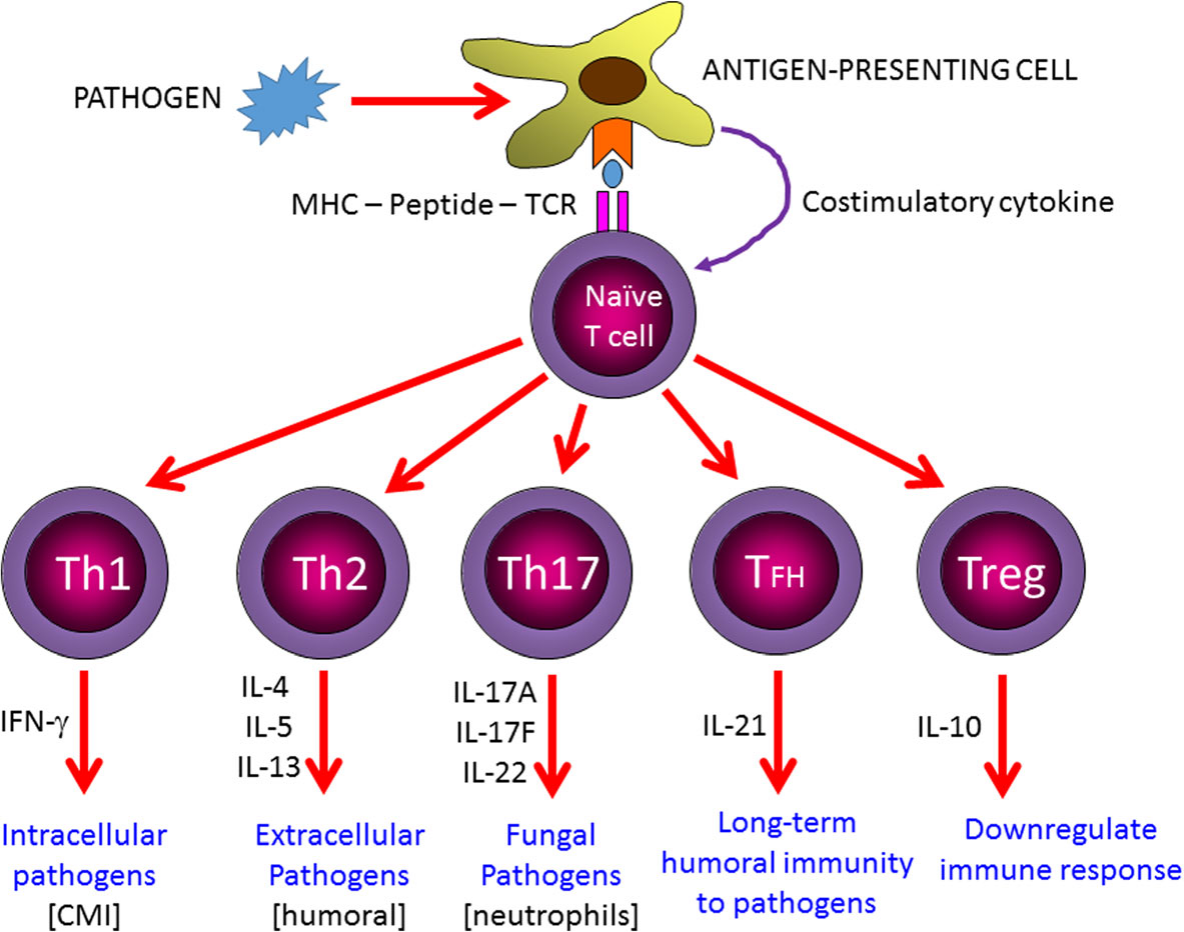
Model of immune response to different classes of pathogens. The pathogen is taken up by an antigen-presenting cell (APC) following interactions between antigenic motifs on the pathogen and pattern recogniton receptors expressed by the APC. The pathogen is processed and pathogen-derived antigenic peptides are expressed on the surface of the APC in association with molecules of the major histocompatibility complex (MHC). The T-cell receptor (TCR) of a naïve T lymphocyte recognizes the MHC-peptide complex and the cell receives costimulatory cytokine and surface molecular signals from the APC. The naïve T cell differentiates down one of the pathways of CD4^+^ T cell development as determined by signalling from the APC. T helper 1 (Th1) cells produce interferon (IFN)-γ and direct cell-mediated immunity (CMI) to intracellular pathogens (e.g. viruses, mycobacteria and many arthropod-borne microparasites). Th2 cells produce interleukin (IL)-4, IL-5 and IL-13 and direct antibody responses (humoral immunity) to extracellular pathogens. Th17 cells produce IL-17A, IL-17 F and IL-22 and respond to fungal infections by mobilizing neutrophils. T follicular helper (T_FH_) cells produce IL-21 and lead to establishment of long-term protective humoral immunity via generation of high-affinity antibodies. In contrast to all of the preceding cells, which have a positive action in antimicrobial defence (effector T cells), regulatory T cells (Treg) produce IL-10 and are responsible for down-regulation of immune responses; sometimes in balance with effector T cells to achieve non-sterilizing immunity allowing an animal to be infected, but without significant clinicopathological effect. The balance between the activity of these cells determines the outcome to infection, and in the context of this review, it might be that dogs and cats have a different balance between these cells within their immune responses

Is it possible to tie all of these elements of immunity and immune response together to model the differences between dogs and cats (Fig. 1)? For example do dogs have an immune system dominated by Th2 immune responses (involving antibody production) that explains their relative susceptibility to allergic diseases and autoantibody-mediated diseases, and are such diseases less common in cats because they mount an opposing Th1 immune response (involving cell-mediated immunity with cytotoxic responses driven by the cytokine interferon [IFN]-γ)? At this level, such a model might work, but it falls down when one considers the relative frequency of viral infections in cats - which should not occur if they had a background of protective Th1 immunity. In the case of arthropod-borne infectious diseases, such a Th1 versus Th2 model might be proposed to explain the dichotomy between cats and dogs. If dogs truly were a Th2-dominated species they might logically have greater susceptibility to vector-borne pathogens that often require a Th1 immune response to control or sterilize the infection.

A difference in immune responsiveness has been proposed for leishmaniosis. It is now well known that the resistance or susceptibility of a dog to *Leishmania infantum* infection and clinical disease is determined by the immune response; and that this is likely under genetic control [87]. A resistant dog mounts a Th1 immune response in which IFN-γ signals infected macrophages to destroy intracellular amastigotes, but Treg-derived interleukin (IL)-10 prevents sterilization of the infection and maintains that dog as a reservoir of *L. infantum*. This control of the infection limits the clinicopathological damage. In contrast, a susceptible dog mounts an inappropriate Th2 immune response in which cytokines such as IL-4 and IL-13 activate B lymphocytes, leading to hypergammaglobulinaemia, autoantibody and immune complex formation. Such dogs develop severe life-threatening clinical disease, largely related to secondary immunopathology [18]. A recent review has proposed that the feline immune response to *L. infantum* may differ from that of the dog. Although some cats develop severe clinical disease following *L. infantum* infection [88], this species is suggested to have a ‘natural immunity’ that often allows spontaneous resolution of lesions post infection due to effective Th1 immunity. However, following such resolution, there is seroconversion and antibody titre has been linked to control of infection with reduced positivity in polymerase chain reaction testing [89]. These possible species differences are intriguing and should be further explored.

### Are there genetic differences between dogs and cats?

Can all of these disease susceptibility and immune function differences between dogs and cats be explained by their relative genetics? The most important genes regulating immune responsiveness are those of the MHC. Dogs and cats are unusual amongst mamallian species in having this gene complex spread over two chromosomes - a break that occurred before the divergence of these species over 55 million years ago [90]. The cat also appears to lack one of the loci within the MHC class II gene cluster (the DQ gene) [90], the implication of which might be that cats have more restricted possibilities for antigen presentation. We know that inbreeding has led to limited genetic diversity within the different breeds of dog [91–94] and that within breeds there is a high linkage disequilibrium (i.e. non-random association of alleles at different loci on chromosomes) and restricted MHC type. This means that the dog is a particularly valuable model for genetic studies of disease. In contrast, there is much less linkage disequilibrium in cats and feline breeds compared with the dog [95]. Such restricted genetic diversity might help explain the susceptibility of dogs to certain diseases, including potentially, the arthropod-borne infectious diseases.

### Are there differences in regulation by the canine and feline microbiome?

It is increasingly recognized that immune development, immune function and susceptibility to disease is regulated by the microbiome, particularly that of the intestinal tract. Particular constituents of the microbiome are powerful inducers of regulatory T cells that control autoimmune and allergic disease, but other organisms or a changed balance in the microbiome (i.e. dysbiosis) might trigger pathological immune reactions within the intestinal mucosa and other organs [96, 97]. Therefore, if immune function is so closely regulated by the microbiome, could differences in canine and feline immunity lie at this level?

Over 20 years ago, it was proposed that dogs and cats had distinct differences in the bacterial content of the small intestine. Cultures of duodenal juice revealed 10^2^ to 10^5^ colony forming units (cfu) of bacteria in the canine proximal small intestine, but 10^5^ to 10^8^ cfu in the equivalent area of the feline intestine [98]. It was suggested that these differences might impact on the relative occurrence of inflammatory enteropathy in the two species. However, more recently we appreciate that such culture techniques were highly inacurate and key differences likely relate to the composition rather than the number of organisms within the microbiome.

Recent studies have begun to characterize the canine and feline intestinal microbiomes. It seems that individual animals have very distinctive and very stable microbial compositions [99], but that differences do exist between dogs and cats, both with respect to the type of organisms and with the metagenomic function (i.e. metabolic profiles) of those organisms [100]. Further investigations have shown broad similarity in the major families of bacteria within the dog and cat microbiome, but cats having much greater diversity in the fungal components of the microbiome relative to dogs [101]. There are also differences between the species in disease; intestinal dysbiosis in canine IBD is characterized by increased representation of *Clostridium perfringens*, but this increase is not seen in the intestinal microbiome of cats with IBD [102, 103].

## Conclusions

Although dogs and cats largely share equivalent immune systems, there are clear differences between the species as to how the elements of the immune system interact – creating species diversity in susceptibility to, and clinicopathological expression of, immune-mediated, neoplastic and infectious diseases. No simple immunological model can summarize these differences in immune function, but immunity might be regulated by distinct genetic backgrounds and potentially by differences in the intestinal microbiome in dogs and cats. If cats are really less susceptible than dogs to arthropod-borne infectious diseases, it remains possible that such resistance relates to differential immune function. However, there are still much simpler explanations that might account for the species difference in occurrence of vector-borne diseases and much work is still required to characterize more accurately the true prevalence and clinical significance of these infections in the cat.

## Abbreviations

cfu: Colony forming units
EBP: Eosinophilic bronchopneumopathy
IFN: Interferon
IL: Interleukin
MHC: Major histocompatibility complex
NOD: Nucleotide oligomerization domain
Th: T helper cell
Treg: Regulatory T cell
